# Noncoding RNAs related to the hedgehog pathway in cancer: clinical implications and future perspectives

**DOI:** 10.1186/s12943-022-01591-z

**Published:** 2022-05-17

**Authors:** Jia Song, Yuexin Ge, Xiaoyu Sun, Qiutong Guan, Shiqiang Gong, Minjie Wei, Jumin Niu, Lin Zhao

**Affiliations:** 1 Department of Pharmacology, School of Pharmacy, China Medical University, Shenyang, 110122 People’s Republic of China; 2 Liaoning Key Laboratory of Molecular Targeted Anti-Tumor Drug Development and Evaluation, China Medical University, Shenyang, 110122 People’s Republic of China; 3 Shenyang Kangwei Medical Laboratory Analysis Co. LTD, Shenyang, 110000 People’s Republic of China; 4 Department of Gynecology, Shenyang Women’s and Children’s Hospital, Shenyang, 110011 People’s Republic of China

**Keywords:** NcRNAs, Hedgehog pathway, Cancer, Biomarker, Targeted therapy

## Abstract

Cancer is a type of malignant affliction threatening human health worldwide; however, the molecular mechanism of cancer pathogenesis remains to be elusive. The oncogenic hedgehog (Hh) pathway is a highly evolutionarily conserved signaling pathway in which the hedgehog-Patched complex is internalized to cellular lysosomes for degradation, resulting in the release of Smoothened inhibition and producing downstream intracellular signals. Noncoding RNAs (ncRNAs) with diversified regulatory functions have the potency of controlling cellular processes. Compelling evidence reveals that Hh pathway, ncRNAs, or their crosstalk play complicated roles in the initiation, metastasis, apoptosis and drug resistance of cancer, allowing ncRNAs related to the Hh pathway to serve as clinical biomarkers for targeted cancer therapy. In this review, we attempt to depict the multiple patterns of ncRNAs in the progression of malignant tumors via interactions with the Hh crucial elements in order to better understand the complex regulatory mechanism, and focus on Hh associated ncRNA therapeutics aimed at boosting their application in the clinical setting.

## Introduction

High mortality and recurrence rates are the major clinical hallmarks of tumors, which gravely harm human well-being and quality of life in the absence of definitive early symptomatic manifestation and efficient medical interventions. Constitutive activation of the hedgehog (Hh) pathway induces the expression of target genes related to cell proliferation, migration, and apoptosis, playing an indispensable role in embryogenesis and organism homeostasis [1]. Some studies have shown that the aberrant activation of the Hh pathway is linked to the formation of some cancers [2–4]. As a result, numerous inhibitors of the Hh pathway are now being investigated for therapeutic application.

Data from Human Genome Project research demonstrates that there are only less than 2% of protein-coding sequences in the human genome with the remaining 98% of the non-coding nucleic acid sequences, generally regarded as useless “garbage” and “noise”, because the vast majority of them are only transcribed into RNAs, and do not continue to translate into functional proteins [5, 6]. However, the latest report launched by the Encyclopedia of DNA Elements project has shown that at least 80% of the genome, notably the remaining “junk” sequences in the human genome, is functional, with the ability to be transcribed into noncoding RNAs (ncRNAs) [7]. Employing high-throughput sequencing technology and bioinformatics methods, researchers have found that a large number of ncRNAs from extensive transcription of non-coding sequences perform important physiological and pathological functions in individuals’ lives, and participate in the progress of some major disorders such as cancer, cardiovascular and neurologic diseases [8–11]. At present, ncRNAs can be loosely divided into circular RNA (circRNA), microRNA (miRNA), long noncoding RNA (lncRNA), small nuclear RNA (snRNA), and so on [12, 13] by length. More and more studies prove that targeting these small molecules is a novel effective modality for cancer treatment, although ncRNAs are initially regarded as “junk” in the genome.

Despite Hh pathway inhibitors, the “off-target effects” and drug resistance hinder the radical cure of malignant tumors. Recently, the molecular mechanism of some ncRNAs regulating protein-coding genes in the tumor-related Hh pathway has been gradually elucidated, which indicates the critical significance of ncRNAs and Hh signaling in cancer pathogenesis and provides clues for the cancer cure and recurrence elimination. This article focuses on ncRNAs, which are prominently expressed in malignancies, to study their molecular regulatory mechanisms in the Hh pathway for cancer therapy, especially circRNAs, miRNAs, lncRNAs, and snRNAs.

### Overview of hedgehog signaling

The Hh pathway serves a crucial purpose in early embryonic development and formation of organs and tissues, but it is dormant in adult tissues. The name “hedgehog” origins from the intrinsic mutation of the Hh gene in the cuticle of Drosophila larvae, which exhibits a spiked appearance resembling that of a frightened hedgehog [14]. In fact, Hh gene was first discovered by Nüsslein-Volhard C and Wieschaus E 40 years ago via genetic screens in Drosophila [15]. Specifically, the Hh gene family is involved in the formation of nervous system, organs, cartilages, and gonads in various vertebrates, which was reviewed by Hammerschmidt M et al. [16]. Its sequence is also isolated from invertebrates such as *Hirudo medicinalis* (leech), *Diadema antillarum* (sea urchin), and cephalochordate amphioxus (between invertebrate-vertebrate transitional stage) [17, 18]. Many human disorders, including congenital malformations, Alzheimer’s disease, diabetes and malignant tumors, are undeniably linked to abnormal Hh protein activity [19–22]. In addition to normal organisms, there is definite evidence that this pathway will be inappropriately activated in some human tumors, as well as related to initiation, invasion, migration, apoptotic cell death, and epithelial-mesenchymal transition (EMT) of malignant cells [1, 23], which could be attributed to the damage repair mechanism of tumor cells.

Hh pathway signal transducers mainly consist of Hh ligand, twelve-transmembrane receptor Patched (Ptch), G protein-coupled receptor Smoothened (Smo), glioma-associated transcription factor (Gli), and other target genes. (1) Three soluble Hh ligands with lipid modification have been found in vertebrates, including sonic hedgehog (Shh), Indian hedgehog (Ihh), and desert hedgehog (Dhh). They have different distributions in different tissues, but all of them can bind to Ptch [1]. The most studied Shh is mostly distributed in the nervous system, skin and digestive tract; Ihh is primarily located in bone and cartilage; Dhh is mainly found in the gonads. (2) In addition, the Hh protein receptor Patched with the 12-transmembrane domain has two kinds of homologs in vertebrates: Ptch1 and Ptch2, with Ptch1 in mesenchymal cells playing a leading role. (3) There are three types of transcription factors, including Gli1, Gli2, and Gli3, which play a positive or negative role at the transcriptional and post-transcriptional levels [24]. Only full-length Gli, on the other hand, may enter the nucleus and trigger Hh target gene expression. (4) Hh potential target genes consist of hedgehog-interacting protein (Hhip) [25], proliferative regulator N-myc proto-oncogene protein (Mycn) [26], cell cycle regulator G1/S-specific cyclin-D1 (CCND1) [27], D2 (CCND2) [28], E1 (CCNE1) [29], apoptotic regulator B-cell lymphoma 2 (Bcl2) [30], EMT related genes snail, twist1 [31] and angiogenic regulator vascular endothelial growth factor A (VEGFA) [32] (Table 1).

**Table 1:** Elements of hedgehog pathway signal transduction

Gene	Full name of gene	Functions	Ref
Shh, Ihh, Dhh	Hedgehog ligand	Positive regulator of the Hh pathway	[1]
Ptch1	Patched 1	Negative regulator of the Hh pathway	[1]
Smo	Smoothened	Positive regulator of the Hh pathway	[33]
Sufu	Suppressed fusion protein	Negative regulator of the Hh pathway	[34]
Gli1, Gli2, Gli3	Glioma-associated oncogene 1, 2 and 3	Positive or negative regulator of the Hh pathway	[24]

Ptch competes with Smo for cholesterol binding due to shared sterol-binding sites, and sufficient cholesterol levels on the cell membrane are required for Smo activation [35–37]. The direct Hh receptor Ptch with cilia localization binds to cholesterol in the absence of the Hh ligand, leading to low cholesterol levels in the membrane. Smo, a crucial signal transducer with cytoplasmic vesicles, is inhibited into cilia and target genes are switched off as a result. In general, suppressor of fused (Sufu), kinesin protein (Kif7), and Gli form the cytoplasmic complex. Full-length Gli is phosphorylated by protein kinase A (PKA), glycogen synthase kinase 3β (GSK3β), and casein kinase 1 (CK1) in the absence of Hh signaling, and enters the nucleus in truncated form as Gli repressor (GliR), keeping downstream target genes expression blocked.

**Fig. 1:**
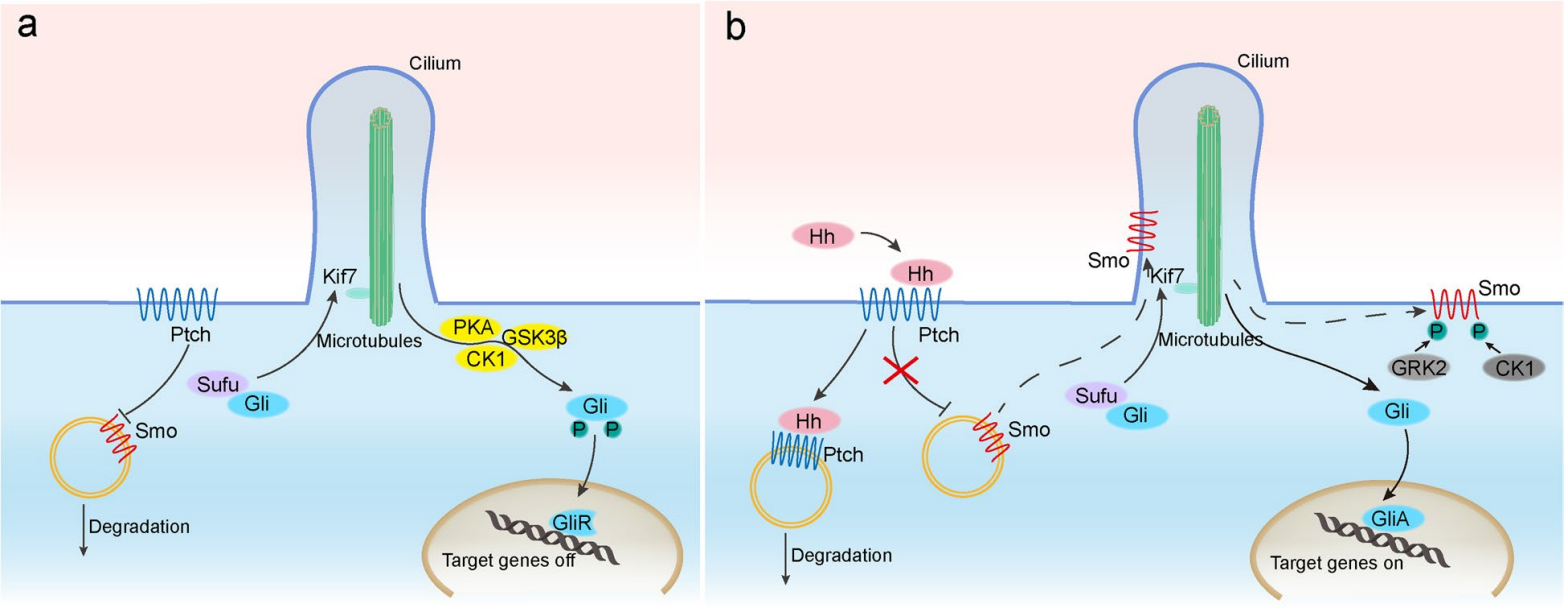
Hedgehog signaling pathway in vertebrates. **a** In the absence of Hh ligand, Ptch inhibits the cilia translocation and activation of Smo. The Gli-Sufu-Kif7 complex forms, and the full-length Gli is subsequently phosphorylated by PKA, GSK3β and CK1 to become GliR with truncated form. The GliR enters the nucleus to inhibit the transcription of Hh target genes. **b** The binding of Hh-Ptch releases the inhibition of Smo. Activated Smo promotes Gli to escape from the bondage of Sufu and free to become GliA in a full-length form, entering the nucleus to induce target gene expression

Ptch moves out of cilia when Hh ligand binds to it. The Hh-Ptch binding complex is internalized and degraded in the lysosomes [38]. The binding also relieves the inhibition of Smo, because Smo is triggered by binding to free cholesterol once it accumulates in the cilia. Activated Smo makes full-length Gli1 release from Sufu to be Gli activator (GliA) migrating into the nucleus, to activate the expression of downstream target genes. Smo phosphorylated by G protein-coupled receptor kinase 2 (GRK2) and CK1 enters the cytoplasm instead of being degraded and endocytosed, and ultimately relays signals to the Gli protein [33, 34, 39–42]. In terms of Smo activation, a recent study indicates that Smo-193a.a. encoded by circSmo can promote Smo cholesterol modification and activation [43], which has guiding significance for unraveling the intricate relationship. It’s worth noting that the regulatory patterns of the Hh signaling pathway can be usually categorized as canonical and non-canonical mechanisms, the latter of which is further divided into two types: Smo-dependent and Ptch-dependent pathways, which are reviewed in detail elsewhere [2]. In addition, the above-mentioned signal transduction mechanism is referred to as the canonical Hh signaling pathway [44] (Fig. 1).

### Hedgehog signaling in cancer

**Fig. 2:**
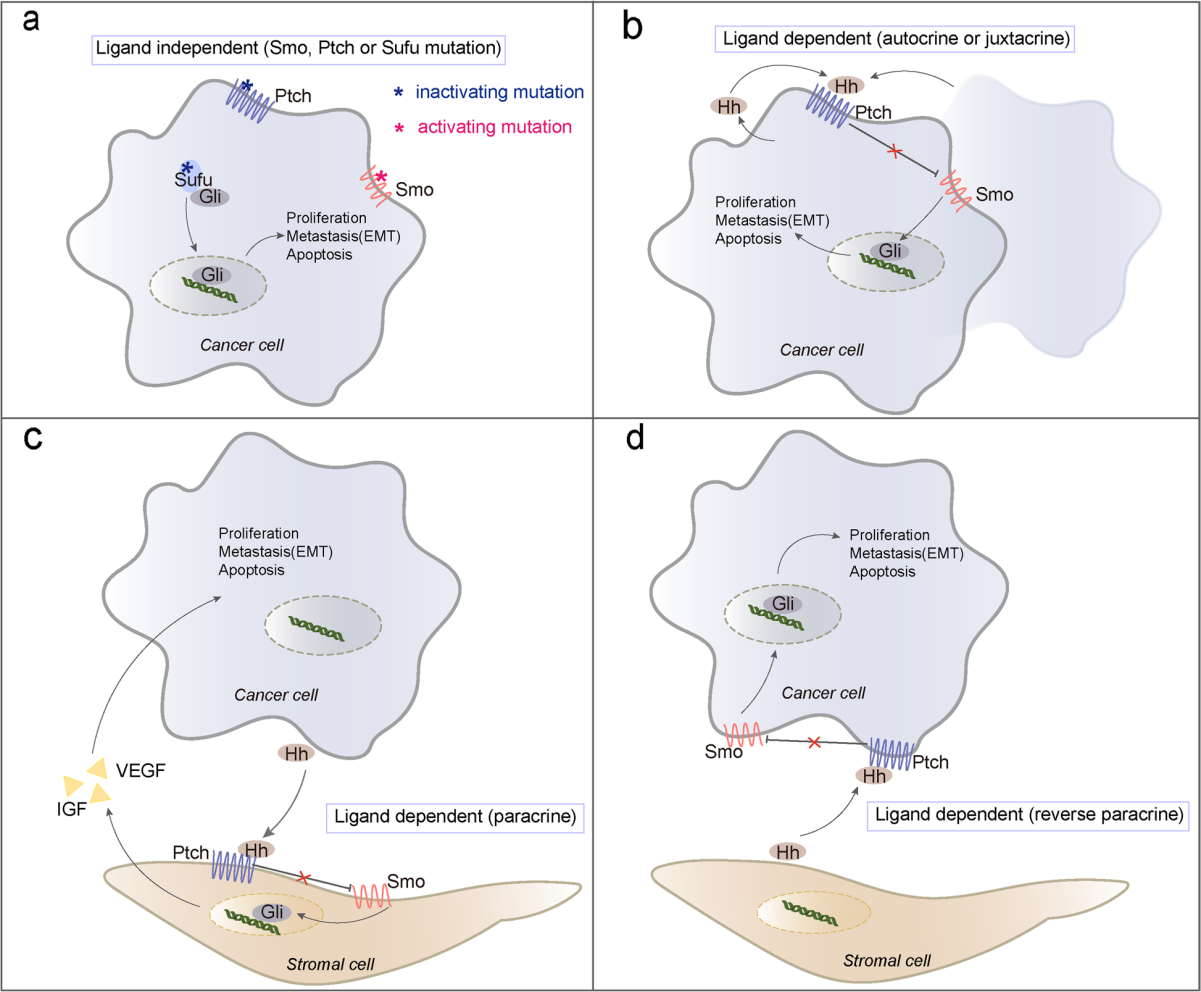
Three patterns of abnormal hedgehog signaling activation in cancer. Positive and negative regulatory components, Smo and Ptch are depicted in red and blue, respectively. **a** Type 1 - ligand independent oncogenic Hh pathway (autonomous). The gain-of-function mutant Smo (red asterisk) and the loss-of-function mutant Ptch or Sufu (blue asterisk) can activate the Hh signaling pathway. **b** Type 2 - ligand dependent oncogenic Hh pathway (autocrine or juxtacrine). The autocrine Hh ligand of tumor cells is taken up by the same tumor cells (autocrine) or adjacent tumor cells (juxtacrine), and stimulates the Hh signaling in cancer by interacting with Ptch and Smo. **c** Type 3/a - ligand dependent oncogenic Hh pathway (paracrine). This is the paracrine mode in which stromal cells take up the Hh ligand secreted by tumor cells, and then secrete some growth factors, such as VEGF and IGF, which provide a suitable environment for tumor cells growth. **d** Type 3/b - ligand dependent oncogenic Hh pathway (reverse paracrine). This is the reverse paracrine mode in which tumor cells directly take up the Hh ligand secreted by stromal cells, triggering cascade response of Hh signaling

The hallmarks of tumors are reflected in uncontrolled cell growth, gene instability, strong self-repair ability, and so on. Currently, one-third of cancers are thought to be correlated with aberrant activation of the Hh signaling pathway. Further investigations have proven that mutant or dysregulated Hh signaling could interfere with tumor behavioral phenotypes, contributing to the onset, growth, metastasis, and apoptosis of pancreatic cancer, lung cancer, ovarian cancer, breast cancer, esophageal cancer, and colorectal cancer [45–50]. It became clear that there are three patterns of eliciting the Hh signaling cascade in multiple cancers [44, 51] (Fig. 2):

### Type 1 - ligand independent oncogenic Hh pathway (autonomous)

Type 1 of Hh pathway activation is caused by activating or inactivating mutations, respectively, in the Smo gene, or the Ptch and Sufu gene, as mentioned in a previous report [52]. The study also systematically elucidated 10 skin cancer-related genes mutation in 42 patients with sporadic basal cell carcinoma (BCC), including Smo, Ptch, and Sufu. Furthermore, the somatic mutation frequency of Ptch in studied tumors was 67% higher than previously reported. The heritable mutation of the Ptch gene in a rare autosomal dominant disease - nevoid basal cell nevus syndrome, commonly known as Gorlin Syndrome [53] was the first example of this sort of genetic mutation. Subsequently, the mutation of Ptch in sporadic medulloblastomas and other cancers was also confirmed [54–56]. In parallel, there are some reports on tumor-activator Smo and tumor-suppressor Sufu mutations in sporadic BCC, medulloblastoma and other tumors [57–59]. Thus, the core regulatory factors - Ptch, Sufu, and Smo gene mutations give us some new insights into the clinical diagnosis and treatment of human malignancies.

### Type 2 - ligand dependent oncogenic Hh pathway (autocrine or juxtacrine)

In addition to the Hh gene mutation, Hh ligand secreted by tumor cells, is also an important factor driving the Hh pathway. Hh ligand is claimed to control malignant behavior in gastric cancer [60] via autocrine regulation, including cell proliferation, which is consistent with the findings of a recent research that prove that the presence of Hh ligand is required for the occurrence of digestive tract tumors [61]. Of course, the autocrine-juxtacrine pattern is also common in a plethora of cancers including prostate cancer, multiple myeloma, gliomas, and colon cancer [62–66].

### Type 3 - ligand dependent oncogenic Hh pathway (paracrine or reverse paracrine)

The Hh ligand released by tumor cells also acts on stromal cells in another mode - the paracrine pattern. By co-culturing tumor cells and adjacent stromal cells, studies have revealed that the Hh ligand produced by tumor cells can be taken up by surrounding stromal cells which can secrete some paracrine signals (such as vascular endothelial growth factor (VEGF), insulin-like growth factor (IGF) and other factors) promoting tumor growth to stimulate the Hh pathway, that is, the paracrine model [33, 67, 68], occurred in ovarian cancer, hepatocellular carcinoma (HCC), prostate cancer, breast cancer and other cancers [3, 69–71]. Up the B- and plasma-cell malignancies lymphoma and multiple myeloma [72], certain tumor cells directly take in Hh ligand secreted by stromal cells in the tumor microenvironment (TME) to maintain formation and survival, a process known as reverse paracrine.

### The role of hedgehog signaling in cancer stem cells

Cancer stem cells (CSCs) have been known to be tumor-initiating cells. In 1997, Bonnet and his colleagues first proposed CSCs via a study on human acute myeloid leukemia [73]. The precise definition of CSCs was proposed by American Association for Cancer Research in 2006 [74], and that is ‘a cell within a tumor that possess the capacity to self-renew and to cause the heterogeneous lineages of cancer cells that comprise a tumor’, which implies the necessity of targeting CSCs in tumor therapy. However, the drug resistance carried by CSCs and TME leads to malignant tumors recurrence and anticancer therapy failure.

The abnormal activity of the Hh pathway in cancer stem cell biology has been recently demonstrated in multiple myeloma [4], colorectal cancer [75], glioma [76], and breast cancer [77, 78], whereas pathway blockade lowers CSC proliferation, metastasis, and EMT. In colon cancer, Hh-Gli signaling drives stem cell survival and expansion. In vivo experiments show that Hh-Gli pathway inhibitors cyclopamine or Smo gene silence could effectively inhibit the recurrence and metastasis of colon cancer [65]. Another study focuses on stromal cells in triple-negative breast cancer (TNBC) to see if anti-stromal therapy may be utilized to develop novel cancer treatment strategies [71]. Researchers find via using the murine M6 allograft model of TNBC that Hh ligand generated by tumor cells activates surrounding cancer-associated fibroblasts, which promotes plastic and chemotherapy-resistant phenotypes of CSCs through FGF5 activation and fibrillar collagen deposition. In the mouse model and clinical trial, Smo inhibitor treatment boosts the sensitivity of tumors to docetaxel, significantly reduces metastasis and prolongs survival. As discussed before, targeting CSCs may provide new insights into the reversal of cancer resistance via the modulation of Hh signaling.

### CircRNAs modulate the hedgehog signaling pathway in cancer

CircRNA is a class of ncRNA molecules with no 5’cap and 3’poly A tail [79], which is covalently closed loops with a single chain, processed by back splicing of precursor RNA [80], with resistance to exonuclease mediated degradation, high conservation, strong stability, tissue specificity [81–84], and regulation of gene expression and malignant progression. CircRNAs can be used as miRNA or protein sponges, as well as participate in mRNA transcription and protein coding [85]. Table 2 lists recently reported circRNAs that influence Hh signaling in tumors.

**Table 2:** Dysregulation of circRNAs related to the hedgehog pathway in cancer

CircRNAs	Function	Mechanism	Target in Hh pathway	Cancer	Role	Ref
circSmo	Proliferation (+)	Encodes Smo-193a.a.	Smo	Glioblastoma	Up	[43]
circDGKB	Proliferation (+); Migration (+); Invasion (+); Apoptosis (−)	As miR-873 sponge	Gli1	Neuroblastoma	Up	[86]
circZNF609	Proliferation (+); Migration (+); Invasion (+); Apoptosis (−)	As miR-15a-5p/15b-5p sponge	Gli2	Hepatocellular carcinoma	Up	[87]
circZNF609	Migration (+)	As microRNA-150 sponge	Gli1	Colorectal cancer	Up	[88]
circSMO742	Proliferation (+); Migration (+); Apoptosis (−)	As miR-338-3p sponge	Smo	Glioma	Up	[89]
circ-STAT3	Proliferation (+); Migration (+); Invasion (+); Stemness (+)	As miR-29a/b/c-3p sponge	Gli2	Hepatoblastoma	Up	[90]
circ_0036412	Proliferation (+)	As miR-579-3p sponge	Gli2	Hepatocellular carcinoma	Up	[91]
circDCAF6	Proliferation (+); Stemness (+)	As miR-616-3p sponge	Gli1	Breast cancer	Up	[92]
circGli1	Migration (+); Invasion (+)	Interacts with p70S6K2 protein	Gli1	Melanoma	Up	[93]
circIPO11	Proliferation (+)	Interacts with TOP1 protein	Gli1	Hepatocellular carcinoma	Up	[94]

### CircRNAs encode a new protein

CircRNA - circSmo from exon 3–6 of Smo gene encodes a novel protein with 196 amino acids (Smo-193a.a.) through the internal ribosomal entry site element. Mechanistically, Smo-193a.a. can bind Smo and enhance cholesterol modification of Smo. Thus, Smo is activated and phosphorylated, which in turn promotes the expression of downstream target genes Gli1, CCND1, and Myc. Functional assays have shown that the high expression of Smo-193a.a is associated with poor prognosis of glioblastoma and positively regulates the Hh pathway, while the low level of Smo-193a.a. can significantly inhibit self-renewal of CSCs and tumorigenesis in mice. This implies that the Smo-193a.a. encoded by cricSmo might be a potential therapeutic target for glioblastoma [43].

### CircRNAs as miRNA sponges

Another paper [86] focuses on the association of circDGKB with the growth of neuroblastoma cells. In this research, in vitro studies clarify that overexpressed circDGKB promotes neuroblastoma cells proliferation, metastasis and tumorigenesis. This is the result of circDGKB as miR-873 sponge positive regulation of the Hh signal pathway via the upregulation of Gli1. In addition, circZNF609 [87], as the miR-15a-5p/15b-5p sponge, promotes HCC cells proliferation, metastasis, and stemness by activating the Hh pathway through the regulation of miR-15a-5p/15b-5p and Gli2 expressions, which has been confirmed in functional tests and in vivo studies. It’s reported that circZNF609 sequestering microRNA-150 plays a role in promoting colorectal cancer progression by upregulating Gli1 expression [88]. Moreover, several circRNAs such as circSMO742 [89], circ-STAT3 [90], circ_0036412 [91] and circDCAF6 [92] could act as miRNA sponges to affect some cancer hallmarks.

### CircRNA-protein interactions

A recent investigation unveils that circGli1 interacts with p70S6K2 protein, which can block the binding of GSK3β with Gli1 and β-catenin, to facilitate Gli1 and β-catenin protein expression, activate hedgehog/Gli1 and Wnt/β-catenin pathways and further drive Cyr61 expression by boosting their downstream gene MYC expression, leading to accelerated cell migration and angiogenesis in melanoma. These results provide strong evidence that circGli1 could be a prospective therapeutic candidate for melanoma metastasis [93]. Further investigation reveals that the highly expressed circIPO11 in HCC drives the self-renewal of liver CSCs and promotes the proliferation of HCC by recruiting TOP1 protein to the Gli1 promoter and triggering Gli1 transcription and Hh signaling pathway [94].

### MiRNAs modulate the hedgehog signaling pathway in cancer

MiRNA is defined as a small noncoding RNA with a length of about 22 nucleotides [95]. It mainly plays a role in regulating gene expression via participating in target mRNA degradation or translation inhibition. MicroRNA was first discovered in 1993 [96, 97], and has gradually been widely studied in recent years. There is evidence that miRNA has been implicated in a variety of physiological and pathological processes, such as tumorigenesis, organ formation, and immune function regulation [98, 99]. Targeting miRNAs for cancer therapy has appeared to become a research hotspot in recent years, based on the relationship between abnormal activation of the Hh signaling pathway and the occurrence and development of malignant tumors. Table 3 lists recently reported miRNAs that regulate Hh signaling in tumors.

**Table 3:** Dysregulation of microRNAs related to the hedgehog pathway in cancer

MicroRNAs	Function	Target in Hh pathway	Cancer	Role	Ref
miR205HG	Proliferation (−); Migration (−); Invasion (−)	Shh	Esophageal adenocarcinoma	Down	[100]
miR-132	Proliferation (−); Invasion (−)	Gli1	Glioma	Down	[101]
miRNA-326	Proliferation (−); Apoptosis (+)	Smo	Glioma; Chronic myeloid leukemia	Down	[102, 103]
miR-361-3p	Proliferation (−);	Gli1, Gli3	Retinoblastoma	Down	[104]
miR-338-3p	Proliferation (−); Apoptosis (+)	Smo	Colorectal carcinoma	Down	[105]
miR-338-3p	Migration (−); Invasion (−)	Smo	Hepatocellular carcinoma	Down	[106]
miR-182-5p	Proliferation (−)	Gli2	Lung adenocarcinoma	Down	[107]
miR-1271	Proliferation (−); Apoptosis (+)	Smo	Multiple myeloma	Down	[108]
miR-506-3p	Migration (−); Apoptosis (+)	Shh	Non-small-cell lung cancer	Down	[109]
miR-873-5p	Proliferation (−); Apoptosis (+)	Gli1	Gastric cancer	Down	[110]
miR-324-3p	Proliferation (−); Invasion (−); Apoptosis (+)	Gli3	Nasopharyngeal carcinoma	Down	[111]
miR-218	Apoptosis (+)	Smo	Gastric cancer	Down	[112]
miR-636	Proliferation (−); EMT (−)	Gli2	Ovarian cancer	Down	[113]
miR-506	Proliferation (−); Apoptosis (+)	Gli3	Cervical cancer	Down	[114]
miR-7-5p	Proliferation (−); Migration (−); Invasion (−)	Gli3	Bladder cancer	Down	[115]
miR-7-5p	Proliferation (−); Invasion (−)	Smo	Gastric cancer	Down	[116]
miR-124	Proliferation (−)	Gli2	Glioma	Down	[117]
miR-150	Proliferation (−)	Gli1	Esophageal carcinoma	Down	[118]
miR-202-3p	–	Sufu	Chronic lymphocytic leukemia	Down	[119]
miR-144-3p	Proliferation (−); Invasion (−); EMT (−); Stemness (−)	Gli2	Gastric cancer	Down	[120]
miR-141-3p	Proliferation (−); Apoptosis (+)	Gli2	Osteosarcoma	Down	[121]
miR-212	Proliferation (+); Migration (+); Invasion (+)	Ptch1	Pancreatic ductal adenocarcinoma; Non-small cell lung cancer	Up	[122, 123]
miR-150	Proliferation (+); Migration (+); EMT (+)	Sufu	Gastric cancer	Up	[124]
miR-9	Proliferation (+)	Ptch1	Glioblastoma	Up	[125]
miR-224	Proliferation (+); Invasion (+)	Sufu	Bladder cancer	Up	[126]
miR-214	Migration (+); Invasion (+);	Sufu	Lung adenocarcinoma	Up	[127]
miR-221	Proliferation (+); Migration (+); Invasion (+)	Hhip	Glioblastoma	Up	[128]

Studies have shown that most miRNAs negatively regulate the expression of target mRNA at RNA or protein levels, so miRNAs can be seen as tumor suppressor genes to participate in the cancer process. MiR205HG is identified as a novel tumor suppressor in esophageal adenocarcinoma and Barrett’s esophagus [100]. The expression of miR205HG is negatively correlated with the Shh ligand of the Hh signaling pathway, according to in vivo and in vitro investigations. However, the overexpression of miR205HG effectively inhibits the expression of Ptch1. Follow-up data reveals that Ptch1 is overexpressed in Barrett’s esophagus, which might be linked to its allelic mutation [129], and further research is needed. The anti-glioma miRNA miRNA-132 [130] is considered as a research target to see how it affects the formation of gliomas. Experimental results show that upregulated miRNA-132 blocks proliferation and invasion of glioma cells by inhibiting transcriptional factor Gli1 expression [101]. In addition, miRNA-326 [102, 103], miR-361-3p [104], miR-338-3p [105, 106], miR-182-5p [107], miR-1271 [108], miR-506-3p [109], miR-873-5p [110], miR-324-3p [111], miR-218 [112], miR-636 [113], miR-506 [114], miR-7-5p [115, 116], miR-124 [117], miR-150 [118], miR-202-3p [119], miR-144-3p [120] and miR-141-3p [121] also act as cancer suppressors and interact with critical Hh signaling regulators (such as Ptch, Smo and Gli1) to regulate tumor progression. However, some up-regulated miRNAs, such as miR-212 [122, 123], miR-150 [124], miR-9 [125], miR-224 [126], miR-214 [127] and miR-221 [128] have been reported in tumors that induce tumor formation and recurrence through Hh signaling cascades. Of course, several miRNAs (such as miR-150) have a dual role in different tumors, which can promote or inhibit the growth of cancer. In this case, a case-by-case analysis of clinical diagnosis and treatment is required.

miRNAs as downstream target genes can also be regulated by Hh signaling in addition to regulating Hh signalings. For example, a recent study shows that the transcription factor Gli2 suppresses miR-124 expression by directly binding to the upstream region of the transcriptional start site for miR-124 and AURKA is the direct target of miR-124. Overall, Gli2 drives the growth and progression of human gliomas via the miR-124/AURKA axis [117].

### LncRNAs modulate the hedgehog signaling pathway in cancer

LncRNA is the largest group of ncRNAs with 5′ cap and 3′ poly (A) tail and contains more than 200 nucleotides in length [131, 132]. Functional investigations reveal that lncRNAs control gene expression at epigenetic, transcriptional and post-transcriptional levels, which manipulate multiple biological processes, and are closely related to human diseases [133–135]. Indeed, lncRNAs play a regulatory role mainly by interacting with proteins or nucleotides [136]. Several lncRNAs are linked with specific signaling pathways [137]. Regardless of whether lncRNA plays a direct or indirect role in the signaling pathway, the interaction between lncRNA and signaling pathway indicates their importance in cellular processes. Here, we will focus on several regulatory mechanisms of lncRNAs at the transcriptional or post-transcriptional level. Recently reported lncRNAs that regulate Hh signaling in tumors are shown in Table 4.

**Table 4:** Dysregulation of lncRNAs related to the hedgehog pathway in cancer

LncRNAs	Function	Mechanism	Target in Hh pathway	Cancer	Role	Ref
LINC-PINT	Stemness (−)	As miR-425-5p sponge	Ptch1	Laryngeal carcinoma	Down	[138]
DIO3OS	Proliferation (−); Migration (−); Invasion (−); Apoptosis (+)	As miR-328 sponge	Hhip	Hepatocellular carcinoma	Down	[139]
GAS5	Apoptosis (+)	As miR-378a-5p sponge	Sufu	Triple-negative breast cancer	Down	[140]
LIFR-AS1	Proliferation (−); Migration (−); Invasion (−)	As miR-197-3p sponge	Sufu	Breast cancer	Down	[141]
LOC101930370	Proliferation (+); Migration (+); Invasion (+)	As miR-1471 sponge	Shh	Breast cancer	Up	[142]
TUG1	Proliferation (+)	As miR-132 sponge	Shh	Hepatocellular carcinoma	Up	[143]
LINC01510	Proliferation (+); Migration (+); Invasion (+)	As miR-335 sponge	Shh	Papillary thyroid carcinoma	Up	[144]
LINC01123	Proliferation (+); Migration (+); Invasion (+)	As miR-516b-5p sponge	Gli1	Osteosarcoma	Up	[145]
NEAT1	Proliferation (+); Migration (+); Invasion (+); Apoptosis (−)	As miR-503 sponge	Smo	Hepatocellular carcinoma	Up	[146]
MIRLET7BHG	Proliferation (+); Migration (+); Invasion (+); EMT (+); Stemness (+)	As miR-330-5p sponge	Smo	Hepatocellular carcinoma	Up	[147]
BCAR4	Migration (+); Invasion (+)	Interacts with PNUTS and SNIP1 proteins	Gli2	Breast cancer	Up	[148]
LINC01426	Proliferation (+); Migration (+); EMT (+)	Interacts with USP22 protein	Shh	Lung adenocarcinoma	Up	[149]
lncHDAC2	Proliferation (+); Stemness (+)	Interacts with HDAC2 protein	Ptch1	Hepatocellular carcinoma	Up	[150]
LINC01106	Proliferation (+); Migration (+); Stemness (+)	Interacts with FUS protein	Gli1, Gli2	Colorectal cancer	Up	[151]
HHIP-AS1	Proliferation (−); Migration (−); Invasion (−); Apoptosis (+)	Interacts with HuR protein	Hhip	Hepatocellular carcinoma	Down	[152]
HOTAIR	Stemness (+)	Regulates Gli2 transcription	Gli2	Renal cell carcinoma	Up	[153]

### LncRNAs as miRNA sponges

A paper reveals a new mechanism of interaction among lncRNA, miRNA and mRNA in tumorigenesis [138]. LncRNA LINC-PINT, as a tumor suppressor gene, targets miR-425-5p which targets Ptch1 of the Hh pathway to modulate laryngeal carcinoma cell stemness and chemoresistance. Several other regulatory networks comprising lncRNA, miRNA, and mRNA have been reported in the Hh-mediated cancer, including DIO3OS/miR-328/Hhip [139], GAS5/miR-378a-5p/Sufu [140], LIFR-AS1/miR-197-3p/Sufu [141], LOC101930370/miR-1471/Shh [142], TUG1/miR-132/Shh [143], LINC01510/miR-335/Shh [144], LINC01123/miR-516b-5p/Gli1 [145], NEAT1/miR-503/Smo [146] and MIRLET7BHG/miR-330-5p/Smo [147].

### LncRNA-protein interactions

Researchers identify a novel lncRNA BCAR4 that directly binds PNUTS and SNIP1 proteins, and activates transcription factor Gli2 expression in a chemokine-dependent manner [148]. In lung adenocarcinoma (LUAD), LINC01426 recruits and binds the USP22 protein to promote Shh protein stabilization, which activates the Hh pathway and promotes LUAD cells proliferation, migration, and EMT [149]. lncHDAC2 interacts with HDAC2 protein (a core component of NuRD complex [154]) which recruits NuRD complex to the promoter of Ptch1, to resist the expression of Ptch1, thus causing abnormal activation of the Hh signaling pathway [150]. LINC01106 has been reported to facilitate proliferation and migration of colorectal cancer cells. Further investigation reveals that LINC01106 located in the nuclear could recruit FUS protein to Gli1 and Gli2 promoters, hence activating Gli1 and Gli2 transcription and promoting colorectal cancer formation [151]. Nevertheless, lncRNA HHIP-AS1 [152] acts as a tumor suppressor in HCC progression, which is attributed to its positive regulation of Hhip mRNA stability in a HuR-dependent manner.

### SnRNAs modulate the hedgehog signaling pathway in cancer

The vast majority of cancers are closely related to the genomic changes of driver factors [155]. Although there have been a lot of investigations on cancer drivers, most of them are about coding genes, with only a handful focusing on non-coding drivers. In 2019, Suzuki H et al. [28] discovered non-coding small nuclear RNA U1-snRNA, revealing a new mechanism of abnormal splicing in cancer and providing new ideas for tumor therapy. Among snRNAs, U1 snRNA is the most abundant type of snRNAs transcribed by RNA polymerase II [156]. The study reported that in about half of Sonic hedgehog-type medulloblastomas, there is a highly recurrent hot spot mutation of A > G at the third nucleotide of snRNA, which is rarely found in other subgroups of medulloblastoma. In parallel, this mutation has a higher incidence in adults and adolescents. The U1 snRNA mutation occurs in the 5’splice site binding region, which changes the preferred base-pairing A-U to G-C, creating a new splicing junction and significantly destroying the original splicing pattern. Mechanistically, alternative splicing mediated by U1 snRNA mutations inactivates tumor suppressor gene Ptch1, promotes the expression of downstream oncogenes (Gli2, CCND2) and stimulates the Hh signaling pathway.

### Clinical features and prognosis of ncRNAs associated with hedgehog signaling

Emerging evidence suggests that many ncRNAs are expected to be biomarkers for early diagnosis of cancer, reliable for predicting the prognosis of patients with cancer. Ectopic expression of ncRNAs in specific tumor tissues or circulating body fluids could be used to monitor the occurrence of neoplasms at an early stage. Previous studies show that the expression of DIO3OS is considerably downregulated in HCC tissues compared to paracancerous tissues [139]. The expression of miR-361-3p in retinoblastoma serum is also significantly downregulated [104]. The prognostic value of ncRNAs for patients with cancer is reflected in the correlation analysis between ncRNAs expression and clinicopathological features. LncRNA HOTAIR overexpression is linked to worse prognosis in patients with renal cell carcinoma [153]. In osteosarcoma, elevated levels of LINC01123 expression are related with advanced pathological staging [145]. .Remarkably, miR-324-3p expression is inversely connected with nasopharyngeal carcinoma metastasis but favorably correlated with survival time [111]. These findings suggest that Hh-related ncRNAs have broad clinical application prospects.

### NcRNAs as promising therapeutic strategies for hedgehog signaling mediated cancer

Therapeutic targeting of Hh signaling, as a vital cellular pathway, represents an attractive approach for the treatment of a plethora of cancers. Unremitting efforts have been made towards the development of small molecule inhibitors such as SMO antagonists-vismodegib [157] and cyclopamine [158], Gli antagonists-GANT61 and GANT58 [159] and additional PTCH inhibitors to block Hh signaling at various sites in recent years. Yet there have been some serious drawbacks of utilizing these Hh inhibitors for the treatment of cancer, which has been discussed in detail by Javed Z et al. [160]. Hence, designing persistent small molecule inhibitors with excessive bioavailability is still a thorny question.

The multiple functional repertoires of such naturally occurring ncRNAs hold promise for the development of a potential treatment option in cancers, employing small interfering RNAs, antisense oligonucleotides together with exosomes, nanoparticles and other delivery systems [161], which have been at the clinical trial phase. Owing to their favorable bioavailability, high specificity and good tolerance, engineered nanoformulations may be a reasonable treatment strategy for the Hh signaling mediated cancer. An in-depth analysis of the experimental data shows that PBAE/si-ciRS-7 nanocomplexes with PBAE material could significantly control the malignant behavior of renal cell carcinoma (RCC) tumors based on the carcinogenic effect of ciRS-7, providing theoretical guidance and effective basis for the treatment of RCC [162]. In line with this, researchers also construct a unique nanoparticle system to deliver anti-metastatic microRNA and conventional cytotoxic chemotherapy agents to colorectal hepatic metastases, showing miR-655-3p and oxaliplatin co-delivery system could present the inhibitory effect on tumors [163]. In addition, the successful use of a known anticancer miR-204-5p [164–166] faces many challenges including limited stability, rapid metabolism and so on. To overcome the above-mentioned drawbacks, silica nanoparticles carrying miR-204-5p and oxaliplatin are synthesized to exert their synergistic anticancer effects, leading to high apoptosis rate and limited growth of cells [167]. A novel dual delivery nanoscale device loaded with miR-345 and gemcitabine [168] is developed to treat pancreatic cancer, which greatly achieves the efficacy of miR-345. Immunohistochemical analysis from the tumor tissues of mice shows such nanocomplexes significantly downregulate Shh and its downstream effector Gli1 in the combined GEM+miR-345 treated group compared with the single gemcitabine-treated group. The encouraging findings imply that the entrance of Hh-related ncRNAs therapeutics into clinical testing is promising. Notably, nanotechnology-based system for targeted delivery of ncRNAs to the organ and cell type of interest has been applied in the field of cancer treatment, but the high cost of preparations might impede large-scale production and widespread application.

Accumulating lines of evidence have suggested that proteins and RNAs [169] can be secreted by exosomes that mediate intercellular communication, foreshadowing that exosomes can be an alternative drug delivery platform. As expected, exosomes could easily cross the biological barriers and deliver small molecules of drugs to specific tissues. For instance, exosomal TUG1 derived from cancer-associated fibroblasts promotes metastasis and glycolysis in HCC through the miR-524-5p/SIX1 axis [170]. Engineered exosomes for delivery of miR-21 and 5-fluorouracil (5-FU) are efficiently transferred into 5-FU-resistant colorectal cancer cells [171]. Combined treatment with 5-FU and miR-21 could significantly improve the anti-tumor effect of 5-FU on HCT-116^5FR^ cells compared with the single treatment of 5-FU or miR-21. Furthermore, co-delivery micelles system targeting miR-29b1 and GDC-0449 (also called vismodegib) might synergistically lower liver injury and improve liver fibrosis in mice [172], which will also grant reliable insights and directions for the development of such Hh associated ncRNA therapeutics.

## Conclusion and future prospects

Accumulating evidence shows that the aberrantly activated Hh pathway confers neoplastic cells the propensity of occurrence, proliferation, and migration, which provides a new clue for researchers to explore better therapeutic strategies and drug targets for malignant tumors. Indeed, the development of drugs targeting the Hh signaling driving factors has undoubtedly opened a new door to the battle against tumors. The recognized Hh pathway inhibitors have been considered as potential therapeutic options for cancer therapy. However, owing to the presence of tumor cells heterogeneity, chemical resistance, and the complex pathway crosstalk, it is often difficult for a single pathway inhibitor, such as vismodegib, to absolutely block tumor cell proliferation. This warns us that it is urgent to design a reasonable drug delivery system and develop drugs that target other potential tumor markers.

Data from dozens of reports has indicated that noncoding RNAs can reduce the chemoresistance and stemness of tumor cells, effectively prolong the survival time of patients and reduce cancer recurrence. These small molecules usually participate in disease regulation by affecting the expression of nearby genes, especially parental genes. The ncRNAs related to the Hh pathway can directly or indirectly regulate the expression of Hh pathway genes through multifaceted molecular mechanisms and physiological processes. For example, LINC-PINT, LINC01123 and circZNF609 sponge special miRNAs to modulate target genes Ptch1, Gli1, and Gli2, respectively [87, 138, 145]. To prevent the recurrence of cancer from the perspective of eradicating tumor cells, it is quite necessary for us to consider these two thorny issues in anti-cancer treatment: (1) drug resistance, and (2) stem cells. It has been found in some cancer stem cell models that the expression of ncRNAs, such as circSmo [43], miR-144-3p [120], and lncHDAC2 [150], can interfere with the stemness and chemoresistance of CSCs or tumor cells. In that respect, the appropriate combination of ncRNA-targeted agents and chemotherapeutic medicines is worthy of consideration.

The regulation mechanism of the Hh pathway is intricate and varies with the type and source of cancers. In some specific cases, several stromal cells secrete some Hh ligands or growth factors to stimulate tumor cells. The paracrine modality of the Hh pathway gives us another new anti-cancer strategy, that is, targeting normal stromal cells that shape the pre-malignant environment. It is reported that, unlike tumor cells, most stromal cells are less likely to exhibit genomic instability, which makes it difficult for them to develop resistance to therapeutic drugs. In addition, the crosstalk of Hh pathway and other pathways has been verified [173]. If diagnostic biomarkers targeting multiple pathways simultaneously can be found, it will be a big step towards curing the malignant diseases that threaten human life. As stated in the study [124], Sufu targeted and down-regulated by oncogene miR-150 promotes the occurrence and metastasis of human gastric cancer through dual activation of Wnt/β-catenin and Hh pathways. Circular RNA circ102004 has carcinogenic effects in prostate cancer. In order to clarify the carcinogenic molecular mechanism of circ102004, western blotting assay is performed to detect the expression of key genes in the Hh pathway. It is found that circ102004 is positively correlated with the expression of P-ERK, P-AKT, P-JNK, JNK, β-catenin, and Gli1. In other words, circ102004 is involved in the regulation of some vital signaling pathways including ERK, JNK, Wnt/β-catenin and hedgehog [174]. However, the molecular pattern of circ102004 regulating various signaling pathways has not been clarified and further research remains needed.

**Fig. 3:**
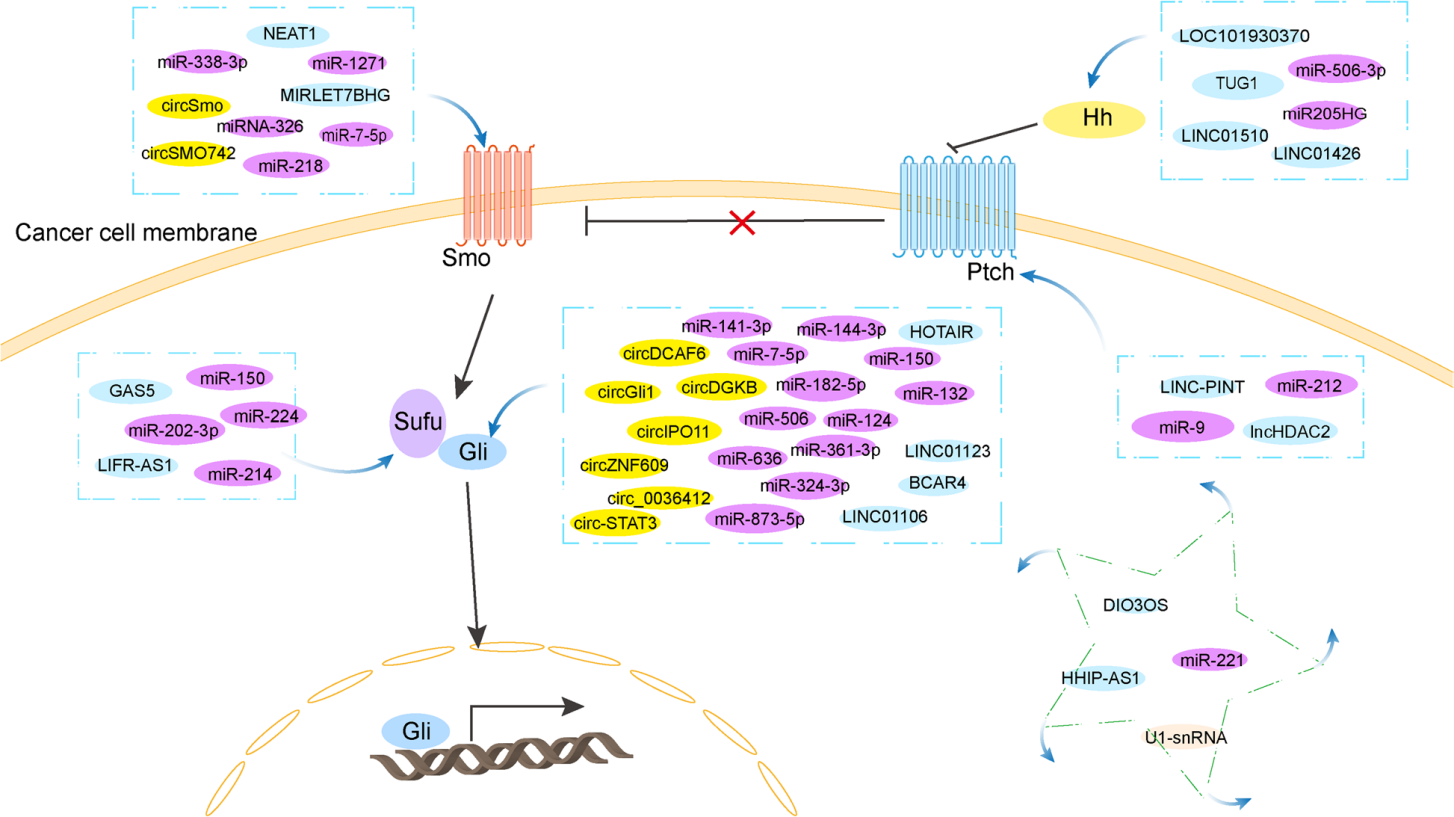
Noncoding RNAs related to the hedgehog pathway in cancer. The five main components of the Hh signaling are Hh, Ptch, Smo, Sufu, and Gli. The ncRNAs introduced in this review include circRNA, microRNA, lncRNA and snRNA. This diagram briefly describes the interaction between ncRNAs and five core components, with the representatives in the pentagram region associated with other Hh-related target genes

“Off-target effects” are major barriers to the development of targeted non-coding RNA technology. The establishment of a reasonable drug delivery system can effectively reduce the failure of treatment caused by “off-target effects”. With the high-end development of pharmaceutical technology, we should consider packaging these small molecules utilizing engineered exosomes or nanoparticles to improve targeting effectiveness. If possible, the designed combination formulation of Hh inhibitors and ncRNAs based on the molecular physical and chemical properties of ncRNAs will be able to extend the time of drug action in vivo, and exert the anti-cancer effect of the medication to the greatest extent. The specific molecular mechanisms of Hh pathway need to be studied as soon as possible, which is also favorable for the successful development and design of targeted drugs. In short, this article summarizes the different roles and molecular mechanisms of ncRNAs in the Hh pathway (Fig. 3), and sheds light on the way in which ncRNAs regulate the Hh pathway, which is conducive to diagnosis, prognostication and treatment of cancer from different angles including protein or RNA levels, and provides a new direction for the clinical therapy of tumors.

## Data Availability

Not applicable.

## Abbreviations

Hh: Hedgehog
ncRNAs: Noncoding RNAs
circRNA: Circular RNA
miRNA: MicroRNA
lncRNA: Long noncoding RNA
snRNA: Small nuclear RNA
EMT: Epithelial-mesenchymal transition
Ptch: Patched
Smo: Smoothened
Gli: Glioma-associated transcription factor
Shh: Sonic hedgehog
Ihh: Indian hedgehog
Dhh: Desert hedgehog
Hhip: Hedgehog-interacting protein
Mycn: N-myc proto-oncogene protein
CCND1: G1/S-specific cyclin-D1
CCND2: G1/S-specific cyclin-D2
CCNE1: G1/S-specific cyclin-E1
Bcl2: B-cell lymphoma 2
VEGFA: Vascular endothelial growth factor A
Sufu: Suppressor of Fused
Kif7: Kinesin protein
PKA: Protein kinase A
GSK3β: Glycogen synthase kinase 3β
CK1: Casein kinase 1
GliR: Gli repressor
GliA: Gli activator
GRK2: G protein-coupled receptor kinase 2
BCC: Basal cell carcinoma
VEGF: Vascular endothelial growth factor
IGF: Insulin-like growth factor
HCC: Hepatocellular carcinoma
TME: Tumor microenvironment
CSCs: Cancer stem cells
TNBC: Triple negative breast cancer
LUAD: Lung adenocarcinoma
RCC: Renal cell carcinoma
5-FU: 5-fluorouracil
